# Comparative Remediation of Arsenic and Antimony Co-Contaminated Soil by Iron- and Manganese-Modified Activated Carbon and Biochar

**DOI:** 10.3390/toxics12100740

**Published:** 2024-10-12

**Authors:** Jiayi Han, Chuang Zhao, Min Yang, Mingheng Ye, Yani Li, Keke Zhou, Junrui Zhang, Peipei Song

**Affiliations:** 1 College of Resources and Environment, Key Laboratory of Agricultural Environment, National Engineering Research Center for Efficient Utilization of Soil and Fertilizer Resources, Shandong Agricultural University, Tai’an 271018, China; 2 Shandong Institute of Geophysical and Geochemical Exploration, Jinan 250013, China; 3 Ministry of Ecology and Environment of the People’s Republic of China, Nanjing Institute of Environmental Sciences, Nanjing 210042, China

**Keywords:** Fe/Mn modification, activated carbon, biochar, arsenic, antimony, soil enzymes

## Abstract

At present, soil contaminated with arsenic (As) and antimony (Sb) is escalating at an alarming rate, which is harmful to human health. In this study, Fe- and Mn-modified activated carbon (AC) and biochar (BC) were prepared and compared for the remediation of As- and Sb-contaminated soil. The effects on the speciation of As and Sb, soil pH, organic matter (SOM), and enzyme activity with various dosages and remediation times were investigated. The results showed that on the whole, the best stabilization effect of As and Sb was achieved with 3% FeMnBC. Furthermore, with increases in time and dosage, the immobilization effect on As and Sb was more significant. Fe/Mn-modified AC and BC enhanced soil pH, with 3% MnAC being particularly effective; 3% AC and 3% FeMnAC demonstrated the most pronounced enhancement in SOM. The modified carbon materials exhibited a dramatic increase in enzymatic activity. In particular, urease activity showed an increasing trend, and catalase activity first decreased and then increased over 30 days. Among the treatments, 3% MnAC showed the most significant enhancements in catalase and urease activities, whereas 1% FeMnBC had the most pronounced effect on increasing sucrase activity. This study provides theoretical support for the remediation of soil co-contaminated with As and Sb by Fe/Mn-modified AC and BC.

## 1. Introduction

Due to their similar sources and chemical properties, arsenic (As) and antimony (Sb) always coexist in the environment [1]. With the acceleration of industrialization, metal mining and smelting, chemical manufacturing, and other factors, As and Sb have been released into the environment in large quantities [2]. As concentrations of Sb in soils across most Asian nations typically range from 5 to 50 mg/kg [3], areas with severe contamination can see these levels increase to between 92 and 840 mg/kg [4]. In uncontaminated environments, Sb concentrations are generally below 1 mg/kg [5], ranging from 0.25 mg/kg to 1.4 mg/kg under natural conditions. However, in several European countries, including Italy, Germany, Switzerland, and France, Sb levels in heavily contaminated soils can reach as high as 1357.10 mg/kg [6]. Due to the large accumulation of As and Sb and long-term exposure, it is harmful to human health, environmental protection, and resource utilization. For example, the soil structure is changed, crop quality and yield are reduced, and contaminated plants enter the food web, resulting in serious damage to biodiversity and the ecosystem [7]. Therefore, effective remediation of soils contaminated with As and Sb has to be paid close attention to and settled urgently.

Physical, chemical, and biological remediation technologies have been widely used in soil remediation [8]; among these, carbon-based materials have attracted wide attention due to their advantages of good stability and abundant sources, and they have been gradually developed as soil remediation materials. Among carbon materials, activated carbon (AC) and biochar (BC) have good effects due to their high porosity and large specific surface area. Li et al. [9] pointed out that activated carbon has a strong adsorption effect on heavy metal ions, such as Zn(II), Cd(II), Ni(II), and Cu(II). Yang et al. [10] found that with 5% fine-stalk biochar, the extraction of Cu and Zn decreased by 97.3% and 62.2% respectively. Silvani et al. [11] found that 20% BC resulted in 61% and 12% reduction in the leaching of Pb and Sb.

However, the effectiveness of activated carbon and BC in the remediation of heavy metal-contaminated soils, especially for oxidizing anionic metals such as As and Sb, still requires a lot of research [12]. Various chemical modifications, such as acidification, oxidation, manganese doping, and iron doping have been used to enhance the oxidative anion-like metal adsorption capacity of AC and BC [13]. For example, iron oxide, with its large specific surface area and rich surface adsorption sites, has a powerful adsorption capacity for heavy metals [14]. Manganese oxide has different properties, such as a low zero-point charge, abundant active sites, and functional groups [15]. Studies have shown that AC and BC modified with Fe or Mn had a larger surface area and a higher adsorption capacity, leading to better stabilization of heavy metals [16,17,18]. To the best of our knowledge, there has been no prior endeavor to employ Fe/Mn-modified AC or BC to remediate soils co-contaminated with As and Sb or compare the effectiveness of Fe/Mn-modified AC and BC.

As a result, this study aimed to remediate As- and Sb-contaminated soil with Fe/Mn-modified AC and BC to study the remediation effect and the effect on soil pH, organic matter, and enzyme activities. It also compared the remediation performance of AC and BC under different modification conditions for different lengths of time and with different material dosage limitations. Overall, this study provides an effective method for the remediation of soil co-contaminated with As and Sb, reveals the potential mechanism of the remediation of Fe/Mn-modified AC and BC through systematic research and in-depth analyses, and provides strong theoretical support for the future development of remediation for soil contaminated with heavy metals, thus promoting its sustainable and healthy growth.

## 2. Materials and Methods

All chemicals were of analytical grade or higher. The nature of AC and BC are shown in Table S1, as displayed in the previous study [19].

### 2.1. Preparation of Materials

The rice straw was pyrolyzed at 500 °C for 4 h and then sieved and immersed in 1 M HCl for 24 h. FeAC was prepared by mixing 250 mL Fe(NO_3_)_3_ and 28 g AC, and the AC and iron mass ratio was 1:10. Then, 1 M KOH was added to the solution until the pH reached 7.5. The solution above was centrifuged, and then the solids were collected after being washed 4 to 5 times with purified water and freeze-dried (−20 °C) and set aside. MnAC, FeMnBC, and FeMnAC were prepared using a method similar to that used for FeAC, replacing Fe(NO_3_)_3_ with Mn(NO_3_)_2_, as well as Fe(NO_3_)_3_ and Mn(NO_3_)_2_. The material dosage was set at 1% and 3%.

### 2.2. Soil Cultivation

The material was mixed with soil and cultured at room temperature, and three parallels were performed for each treatment. According to a previous study [20] and the detection results, we kept the moisture level at 70% of the saturated water content for static incubation, and samples were taken at 10, 20, and 30 days, respectively, for further analysis. CK stands for untreated soil, i.e., the natural soil.

### 2.3. Determination

Wenzel’s five-step method [21] was used to extract different forms of As and Sb. The pH of the soil was measured with a pH meter with a water-to-soil ratio of 1:2.5, and the soil organic matter (SOM) was measured using the boiling water heating method. A quantity of 0.25 g of air-dried soil samples were passed through a 0.149 mm sieve, weighed, and mixed with 5 mL of 0.2 M potassium dichromate solution and 5 mL of concentrated sulfuric acid. The mixture was shaken evenly and incubated at 100 °C for 30 min for determination. Urease, catalase, and sucrase were detected by indophenol colorimetry, potassium permanganate titration, and 3,5-dinitrosalicylic acid colorimetry, respectively, which are commonly used to determine specific enzyme activities [22].

### 2.4. Statistical Analysis

Statistical significance was analyzed through analysis of variance (ANOVA) using SPSS 25.0, and the significance level was set at *p* < 0.05.

## 3. Results and Discussion

### 3.1. As and Sb Speciation

**Figure 1:**
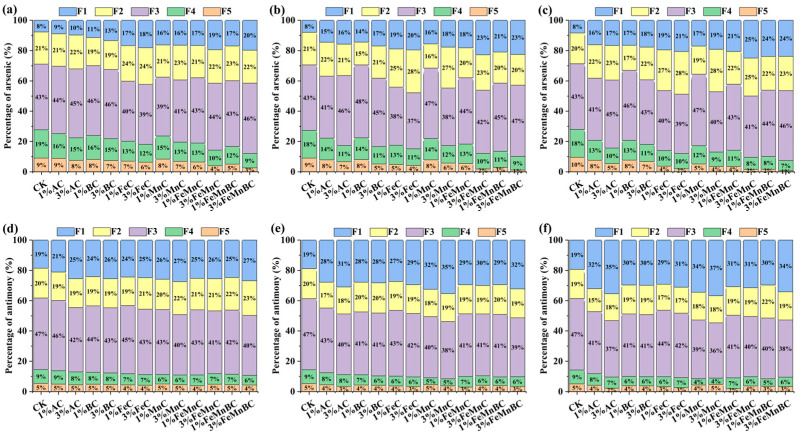
Changes in As speciation with different materials after 10 (**a**), 20 (**b**), and 30 days (**c**) and Sb speciation with different materials after 10 (**d**), 20 (**e**), and 30 days (**f**). (Note: F1: Residue state. F2: Crystalline iron-aluminium oxide bound state. F3: Amorphous iron-aluminum oxide bound state. F4: Specialized adsorption state. F5: Non-specific adsorption state).

The speciation of arsenic (As) and antimony (Sb) can be examined in Figure 1. Compared with Figure 1a, both F1 and F2 were enhanced, while F4 and F5 gradually declined and reached their lowest point, and F3 remained stable, except for 1% MnAC, which increased by 8%. This means that after 30 days with different proportions of Fe/Mn-modified dosing, the stabilization of As increased, and the mobility decreased. The Sb form of the soil showed the following patterns: F4 and F5 were almost unchanged, F2 and F3 decreased, and F1 increased significantly (Figure 1).

Compared with the control, 3% FeMnAC and FeMnBC reached maximum levels in F1, and they reached their lowest percentage after 30 days of treatment in F5 (Figure 1c). This showed that 3% FeMnBC had the best efficiency in stabilizing As and lowering its mobility. In addition, 3% FeMnBC had an adsorption passivation effect on As in soil. This was due to the fact that BC as a loading material provided a good support structure for iron and manganese, which offered more active sites for adsorption [23]. Also, Mn oxidized As(III) into As(V), while Mn(IV) was reduced to Mn(II) and Mn(III), which changed the surface state of Mn oxides and generated new adsorption sites, resulting in stronger adsorption and an increased sequestration capacity. Furthermore, the reaction of Fe(II) and Fe(III) produced secondary iron minerals, which was conducive to As stabilization [24]. In addition, BC effectively reduced Fe and Mn oxide agglomeration and endowed more interfacial active sites.

As shown in Figure S1, the X-ray powder diffraction (XRD) patterns of AC, BC, FeMnAC, and FeMnBC were detected. Due to the diffraction stages of Fe_2_O_3_, the spectrum revealed characteristic reflections at 35.1° [25]. Fe_3_O_4_ was found in both FeMnAC and FeMnBC, and FeMn carbides also occurred in FeMnAC. The peaks at 29.4° and 40.4° revealed the presence of MnCO_3_ and MnO. Fe-Mn oxides are the dominant bimetal crystal [26], indicating that the metal components in the modified biochar provide binding sites for As, Sb, and other organic matter, as well as a foundation for redox reactions [27].

As shown in Figure 1d–f, 3% MnAC displayed the most effective solidification and stabilization performance, with the highest levels consistently observed in F1. This may be due to the two pathways of Sb oxidation by Mn [28], which could play a more important role in the case of Mn-modified AC. The effect of 3% FeMnBC was similar to that of 3% AC, and the content of F1 was the second highest (Figure 1d,f). Modified carbon materials exhibited a stabilizing effect by altering the speciation of Sb, thus facilitating its immobilization [29]. As shown in Table S2, compared with other studies, this work displayed a better immobilization effect on soil co-contaminated with As and Sb in a short time [30,31,32,33,34].

According to the subsequent analysis of soil pH and organic matter, modified BC negatively correlated with soil pH, indicating a decreasing trend. Soil pH is crucial in controlling the speciation, dissolution rate, and mobility of heavy metals in the soil. Modified BC influenced soil pH and the positive charge on colloidal surfaces, ultimately promoting the transformation of anionic Sb speciation through electrostatic attraction [35].

Both As and Sb have the potential to react with Fe and Mn in modified biochar, engaging in electrostatic attraction, complexation, and precipitation reactions in their anionic forms [36,37]. Previous studies have revealed that biochar contains various functional groups, such as “-CH”, “O-H”, “C=C”, “C=O”, etc., which can bind with Fe and Mn [38], thereby enhancing its capacity to bind with As and Sb. As and Sb can be complex, with the oxygen-containing functional groups in modified biochar, further forming As_2_O_3_ or As_2_O_5_ [39,40]. Additionally, Sb can undergo redox reactions, converting between Sb(III) and Sb(V) [41]. Sb forms Sb-O complexes, and Sb(V) can coprecipitate with certain Fe(III) minerals [42].

### 3.2. Soil pH and Organic Matter

**Figure 2:**
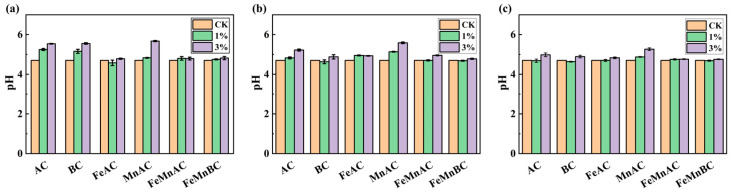
Changes in soil pH with different materials after 10 (**a**), 20 (**b**), and 30 days (**c**).

As shown in Figure 2, the addition of modified carbon materials resulted in temporal pH variation characterized by an initial rise followed by a decrease. Soil pH climbed due to three factors, including alkaline ions from modified carbon materials [43], OH^−^ generated by manganese oxidation, H^+^ consumed by iron oxidation, and organic functional groups like -COO- and -O-, together with carbonate species in biochar. Following their application to the soil, the alkaline moieties in BC and AC were rapidly discharged, neutralizing soil acidity and consequently elevating the soil pH [44]. With the extension of treatment time, soil pH decreased due to the consumption of alkaline base ions in modified carbon materials, and the impact of modified carbon material application on the soil pH buffering capacity was small [45]. A dosage of 3% of these materials typically yielded a significant increase in pH. The pH enhancements induced by AC and BC were quite similar, while FeAC, FeMnAC, and FeMnBC exhibited a smaller impact on pH. Compared with AC, the pH elevation induced by FeAC, FeMnAC, and FeMnBC was diminished. This was due to the specific properties introduced by the iron and manganese modifications, which affected the buffering capacity or altered the ionic interactions within the soil matrix, leading to a differential pH response compared with unmodified AC [46]. Due to the release of a significant amount of H^+^ through the hydrolysis of iron, the elevation of soil pH by FeMnBC, FeMnAC, and FeAC was less pronounced compared with their unmodified counterparts. However, MnAC had a stronger positive effect than AC. This phenomenon could be attributable to the oxidation of Mn, resulting in the production of OH^−^ in the soil.

Iron undergoes redox reactions in soil environments, influencing pH levels [47]. By interacting with soil components such as H^+^ and OH^−^, iron modified the acid–base equilibrium, playing a role in pH regulation [48]. With their notable cation exchange capability, iron oxides efficiently adsorb acidic cations—predominantly hydrogen and aluminum ions—from the soil solution, helping counteract the deleterious effects of excessive acidity on soil pH [49]. Likewise, manganese participates in redox processes within soils, impacting pH values [50]. Specifically, manganese’s oxidation generated alkaline byproducts, increasing soil pH. When modified carbon materials were added to soil, manganese oxides were slowly released into the soil matrix. Owing to their naturally basic properties, these manganese oxides contributed to a further rise in soil pH [51].

**Figure 3:**
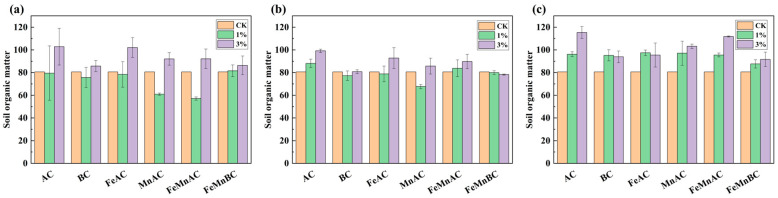
Changes in soil organic matter with different materials after 10 (**a**), 20 (**b**), and 30 days (**c**).

As shown in Figure 3, after the addition of carbon materials, the soil organic matter (SOM) with a 1% dosage initially decreased before showing an upward trend, whereas the SOM with a 3% dosage exhibited a pattern of increase. This was because of their high surface area and porous structures, which provided numerous sites for nutrient adsorption. Moreover, carbon materials could reduce nutrient leaching by binding nutrients within the soil matrix [52]. The enhancement of SOM was positively correlated with the dosage of carbon materials. AC demonstrated better efficacy in improving SOM than BC owing to its stronger adsorption capacity for SOM [53]. The improvement in SOM by FeAC, FeMnAC, and MnAC was inferior to that of AC, as Mn and Fe were adsorbed onto the pore structure of AC, thereby reducing its capacity to adsorb organic molecules and decreasing the synthesis of SOM. Initially, due to the increase in the specific surface area of BC by manganese oxide, FeMnBC was anticipated to adsorb more organic molecules, leading to a greater enhancement of SOM compared with BC. However, the improvement in SOM by 3% FeMnBC was found to be nearly identical to that achieved by 3% BC because the adsorption of Mn and Fe within the pore structure reduced the BC’s porosity. As a result, this inhibited the ability of FeMnBC to adsorb soil organic molecules, thereby hindering the synthesis of SOM.

### 3.3. Enzyme Activity

#### 3.3.1. Urease Activity

**Figure 4:**
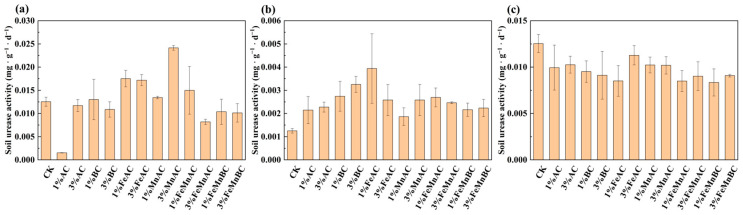
Changes in urease activity with different materials after 10 (**a**), 20 (**b**), and 30 days (**c**).

Figure 4 shows that after 10 days, compared with the control, urease activity significantly decreased with 1% AC, which was possibly related to the fact that urease had the lowest protein solubility at this point [54]. Urease activity increased with 1% FeAC, while it declined with 1% AC, which was due to that Fe enhanced the surface properties of the material and increased the ability to adsorb As and Sb [55]. There was no significant change in urease activity after 20 days. However, after 30 days, it showed an overall upward trend; this was because the soil pH increased, leading to the more stable speciation of As and Sb [56], and the immobilization was positively correlated with urease activity [57]; finally, the activity of urease increased.

#### 3.3.2. Catalase Activity

**Figure 5:**
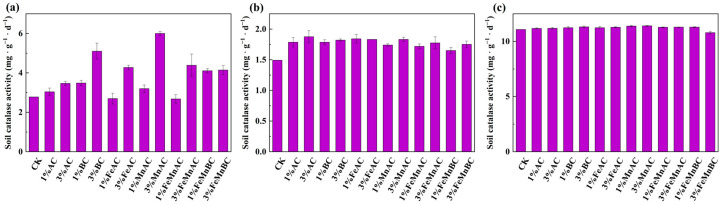
Changes in catalase activity with different materials after 10 (**a**), 20 (**b**), and 30 days (**c**).

It can be seen in Figure 5 that after 10 days, compared with the control, the hydrogen peroxide activity decreased slightly with 1% FeAC and 1% FeMnAC, while it increased significantly with 3% BC and 3% MnAC. Notably, 3% MnAC resulted in an activity level nearly twofold higher than that of the control group. Combined with Figure 3a, 3% MnAC addition elevated the pH to near the optimal range for catalase activity, consequently enhancing catalase activity [58,59]. After 30 days, the immobilization effect of As and Sb improved, as evidenced in Figure 1, thereby reducing the stress on catalase and resulting in an increase in catalase activity to its maximum level.

#### 3.3.3. Sucrase Activity

**Figure 6:**
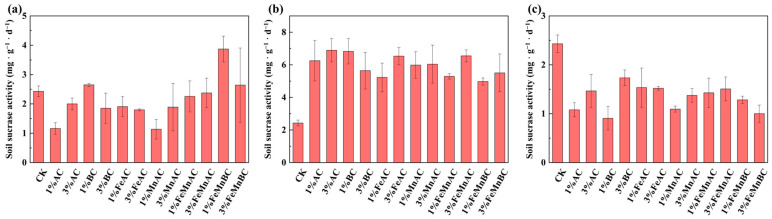
Changes in sucrase activity with different materials after 10 (**a**), 20 (**b**), and 30 days (**c**).

It can be seen in Figure 6 that compared with the control, the activity of sucrase was the highest after 10 days with 1% FeMnBC. On the one hand, it was because the soil enzymes were mainly adsorbed on the soil organic matter in the form of physical or chemical bonding, which changed the spatial structure and activity of the enzyme and avoided the influence of external adverse conditions [60]. On the other hand, the addition of 1% FeMnBC increased organic matter content, which subsequently enhanced soil fertility and elevated sucrase activity [61,62]. This activity continued to rise for approximately 20 days; however, since sucrase is involved in the decomposition of organic matter, an excess of organic matter ultimately inhibited sucrase activity, leading to a pattern in which sucrase activity initially increased and then decreased.

## 4. Conclusions

This work investigated the efficaciousness of Fe/Mn-modified activated carbon (AC) and biochar (BC) in the remediation of soil co-contaminated with arsenic (As) and antimony (Sb). The effects of various dosages and remediation times on the speciation of As and Sb in soil, pH, soil organic matter (SOM), and the activities of its enzymes were explored. The results showed that 3% FeMnBC had an efficient stabilization effect on As and Sb. Mn had an oxidizing effect on As, which made As more stable, and Mn generated new adsorption sites. Fe produced secondary iron minerals, which were conducive to As and Sb stabilization. Fe/Mn-modified AC and BC affected soil pH; this was attributed to the specific properties conferred by the iron and manganese modifications, which potentially adjusted the soil’s buffering capacity and ionic interactions within its matrix, leading to a distinct pH response compared with unmodified AC and BC. The findings demonstrated that 3% MnAC was the most effective in elevating soil pH due to the generation of OH- from the oxidation of Mn. AC outperformed modified AC and BC in enhancing soil organic matter. This superiority was due to Mn and Fe adsorbing onto the pore structure of AC, which reduced its ability to adsorb organic molecules, influencing the synthesis of SOM. In terms of enzyme activity, urease activity showed an increasing trend, and 3% FeMnAC sustained an improvement in urease activity. During the treatment period of 30 days, catalase activity displayed a trend of increasing and then decreasing. After 10 days, 3% MnAC led to the peak activity of catalase. Under the same treatment time, the sucrase activity with 1% FeMnBC treatment increased with the increase in SOM. Overall, these findings prove that 3% FeMnBC is a promising and efficient material for the treatment of soil co-contaminated with As and Sb. In this study, we provide a theoretical basis for the remediation of soil co-contaminated with As and Sb, hoping to offer a new vision for modified carbon composites. In addition, the long-term stability needs to be further studied to ensure its safe application.

## Data Availability

Data will be made available upon request.

## Supplementary Materials

The following supporting information can be downloaded at: https://www.mdpi.com/article/10.3390/toxics12100740/s1, Figure S1: X-ray powder diffraction pattern spectra; Table S1: The nature of AC and BC; Table S2: The comparisons of the relevant studies.
