# Supplementary material for: Comparative Remediation of Arsenic and Antimony Co-Contaminated Soil by Iron- and Manganese-Modified Activated Carbon and Biochar
Source: Toxics. 2024 Oct 12;12(10):740. doi: 10.3390/toxics12100740 (PMC11511182; doi:10.3390/toxics12100740)
Supplement: Supplementary file 1 [file toxics-12-00740-s001.zip › toxics-3210414-supplementary.pdf]

Table S1 The nature of AC and BC

	**pH**	**Surface area ($m^2 \cdot g^{-1}$)**	**C (wt.%)**	**O （wt.%)**	**H (wt.%)**	**N (wt.%)**
AC	7.05	871.0	82.15	3.708	0.939	0.47
BC	9.89	15.7	67.71	7.61	2.178	1.44

Table S2 The comparisons of the relevant studies

Materials	Condition	As immobilization (mg/kg)	Sb immobilization (mg/kg)	References
Fe@H_2O_2-BC (Fe/H_2O_2 modified biochar)	pH 4.57<br>1.5% dosage<br>365 days	201.35	286.72	[30]
A-SCH (Biosynthesis of schwertmannite by Acidithiobacillus ferrooxidans)	5% A-SCH<br>pH from 7.38 to 6.60	196.64		[31]
nZVI@BC (biochar-loaded nanoscale zero-valent iron)	5% dosage<br>30 days<br>nZVI:BC=3:1	26.70		[32]
MBC (manganese oxide-modified biochar composites)	2% dosage<br>100 days	24.69		[33]
BC	1.5% dosage<br>6 months	53.87	2533.03	[34]
FBC (Fe-functionalized biochar)	1.5% dosage<br>6 months	46.68	2000.26	
FeMnBC	pH 4.7<br>3% dosage<br>30 days	123.31	874.28	This study

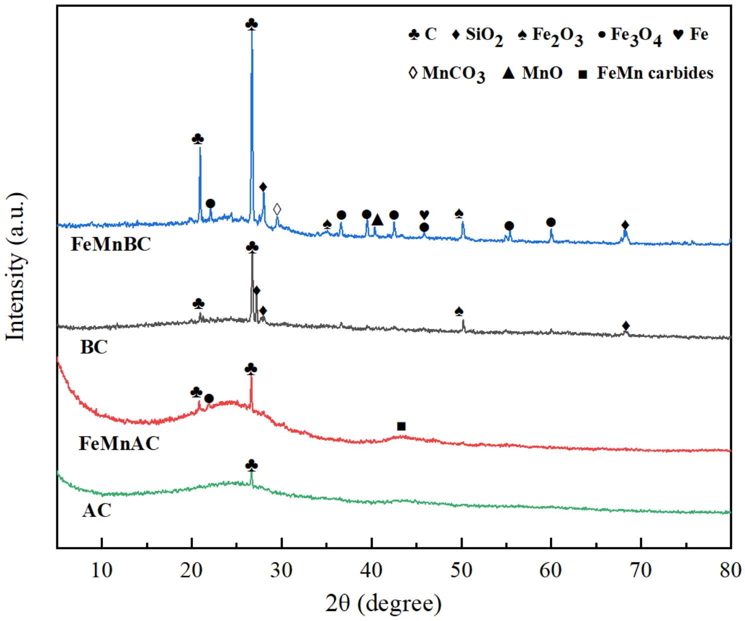

Figure S1 X-ray powder diffraction pattern spectra.
